# Large-voltage behavior of charge transport characteristics in nanosystems with weak electron–vibration coupling

**DOI:** 10.3762/bjnano.6.188

**Published:** 2015-09-03

**Authors:** Tomáš Novotný, Wolfgang Belzig

**Affiliations:** 1 Department of Condensed Matter Physics, Faculty of Mathematics and Physics, Charles University in Prague, Ke Karlovu 5, CZ-12116 Praha 2, Czech Republic; 2 Fachbereich Physik, Universität Konstanz, D-78457 Konstanz, Germany

**Keywords:** electron-vibration interaction, full counting statistics, large-voltage behavior, molecular electronics

## Abstract

We study analytically the Full Counting Statistics of the charge transport through a nanosystem consisting of a few electronic levels weakly coupled to a discrete vibrational mode. In the limit of large transport voltage bias the cumulant generating function can be evaluated explicitly based solely on the intuitive physical arguments and classical master equation description of the vibration mode. We find that for the undamped vibrational modes mutual dynamical interplay between electronic and vibronic degrees of freedom leads to strongly nonlinear (in voltage) transport characteristics of the nanosystem. In particular, we find that for large voltages the *k*-th cumulant of the current grows as *V^2k^* to be contrasted with the linear dependence in case of more strongly externally damped and thus thermalized vibrational modes.

## Introduction

The study of inelastic effects in transport through nanostructures, in particular in molecules [1–3] or atomic wires [4–5] has been an active field of research in past decade. The well-established inelastic electron tunneling spectroscopy (IETS) concept [6] has been applied successfully to single-molecule junctions and provides directly their vibrational frequencies from the position of IETS signals [4,7] and, indirectly from the IETS features such as heights of the peaks, also information about electronic and structural properties [8–9].

Theoretical modeling of IETS signals usually proceeds via combination of ab initio structural density functional theory (DFT) calculations determining the parameters of an effective electron–vibrational Hamiltonian with the non-equilibrium Green’s functions (NEGF) evaluation of the IETS features [10]. It had turned out that in many cases the electron–vibrational couplings are rather weak (dimensionless coupling constant on the order of a few per cents) so that full NEGF calculations typically based on the quite demanding self-consistent Born approximation [11] are not necessary and a computationally less expensive method called the lowest order expansion (LOE) was developed [12–14].

In its original form LOE only works under the assumption of (externally) equilibrated vibrational modes. While this assumption is often satisfied, there are many other cases when it is broken, e.g., in one of the pioneering experiments [4] signatures of the vibrational mode heating were clearly identified [11]. Apart from a full NEGF description in terms of coupled electronic and vibrational Green’s functions [15–16] necessary for intermediate coupling, a simple rate equation for the occupation of the vibrational mode(s) was developed and successfully employed to IETS current in the weak coupling regime [11]. However, this method of mean-field-like accounting for non-equilibrium vibrational occupation cannot be used directly in the calculation of higher-order current cumulants starting with the electronic current noise since it neglects important correlation effects between the electronic and vibronic degrees of freedom, which are relevant even in the weak coupling regime [17]. After realizing this issue there have been several attempts to address this problem within a LOE-like type of formalism, which would be valid in the weak coupling regime only but which would use simplified (semi)analytical formulas for the IETS features with non-equilibrated phonons.

It turns out that there are two methods yielding inconsistent results, namely a paper by Urban et al. [18] and a study by us [19] for the inelastic current noise extended by Utsumi et al. [20] to all current cumulants. In particular, these two approaches disagree (not only but also) in the behavior of the large-voltage asymptotics of current cumulants predicting microscopically for a simplest single-level model different power-laws. Since both approaches address theoretically, via microscopic NEGF formalism, the very same model, the only possibility is that at least one is not correct. Their full detailed comparison is, however, not so easy and straightforward and we thus use an indirect approach which adopts an alternative evaluation of the large-voltage asymptotics to check the validity of the microscopic results. Limit of large voltage, although not so frequently studied as, e.g., close-to-equilibrium linear response, allows for significant simplifications which may lead to full analytical solutions of some problems, such as in these examples [21–23]. Here, we show that also the model of electronic transport through a nanostructure with weak electron–vibrational interaction can be solved exactly in the large-voltage limit with the input of only a few microscopically-derived parameters which can be evaluated (semi)analytically. The solution is not only interesting as a valuable benchmark of full-fledged non-equilibrium microscopic theories and as yet another instance of an exactly solvable model in the large-voltage limit, but it can be with appropriate caution and care extended and applied to realistic experiments measuring higher-order cumulants of inelastic current such as [24].

## Model

The system we consider can be schematically represented as a central device region (representing the molecule or the atomic-wire) which is tunnel-coupled to non-interacting metallic leads

[1]
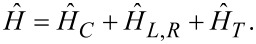


Neglecting for simplicity the spin degree of freedom (based on a spin-less model, our results need to be multiplied by a factor of 2 when compared with works where spin degeneracy is explicitly taken into account) the central region can be described by the following Hamiltonian [10,25]

[2]
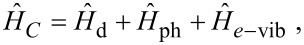


[3]
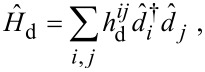


[4]
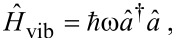


[5]
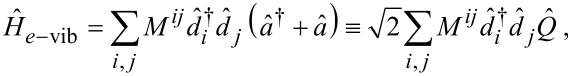


where 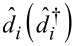 and 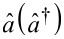 are the electron and vibrational annihilation (creation) operators, respectively; 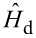 is the single-particle effective Hamiltonian of the electrons moving in a static arrangement of atomic nuclei, 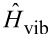 is the Hamiltonian of a free oscillator mode with frequency ω and dimensionless coordinate 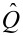, 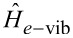 is the electron–vibrational coupling within the harmonic approximation with the coupling matrix *M^ij^*. The leads and tunneling Hamiltonians are given by

[6]
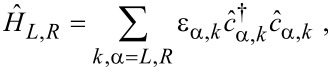


[7]
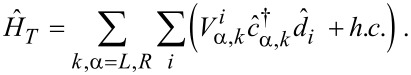


The states in the leads are occupied according to the Fermi distributions *f*_α_(ε) = *f*(ε − μ_α_), with *f*(ε) = (1 + *e*^βε^)^−1^, β = 1/*k*_B_*T* the inverse temperature, and μ_α_ = *E_F_* ± eV/2 the chemical potential of lead α. The applied bias voltage is *eV* = μ*_L_* − μ*_R_*. Tunnel coupling densities are assumed to be energy-independent and read


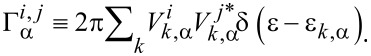


It should be noticed that this effective tight-binding Hamiltonian implicitly contains the effects of the electron–electron Coulomb interaction via the Hamiltonian parameters derived from the mean-field or density-functional theory treatment of the system—for a general strategy see [10]. Since the method is aimed at treatment of “open” central regions (large electronic coupling to the leads quantified by Γ’s), there are expected no strong correlation effects associated with the local Coulomb interaction which would have to be addressed via explicit Hubbard-like Coulomb term(s).

## Double coarse-graining procedure for the oscillator dynamics

Now, we turn our attention to the properties of the system in the limit of large bias voltage *V*. We focus for now on the simplest single-electronic-level model with 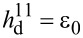, Δ ≡ ε_0_ − *E_F_*, *M*^11^ = *M* and 
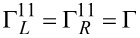
, generalization to the multilevel case will be discussed later on. The reasoning for the large-voltage asymptotics proceeds in two stages of progressive coarse-graining procedure. First, we consider the inelastic (i.e., induced by the electron-vibration coupling) correction to the *mean current.* As shown in Equation 4 and Equation 5 of [26], the large-*V* behavior of the current from the fully microscopic description in terms of non-equilibrium Green’s functions can be reproduced by the following coarse-graining approach. For 
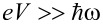
 the characteristic time of electron tunneling across the nanosystem *h*/*eV* is much shorter than the period of oscillation 2π/ω of the vibrational mode and, thus, the oscillator may be considered to be adiabatically gating the single electronic level and consequently changing the electronic transmission coefficient


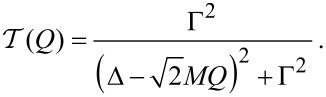


The mean current then results from the averaging the oscillator position *Q*(*t*) = *A* cos(ω*t*) over the oscillation period 2π/ω. The first non-zero correction stems from the second order in *M* expansion of the expression for 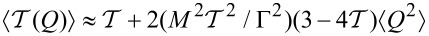 (with the *elastic transmission coefficient* 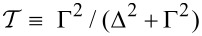), which corresponds to a rectification/ratchet effect due to the nonlinearity of the transmission coefficient in energy. Performing the average 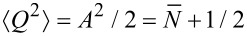 with 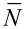 the mean occupation number of the oscillator. This completes the first stage of the coarse-graining scheme, namely, the evaluation of the mean current (we assume that Γ *>> eV* is the largest energy scale and, consequently, neglect the energy dependence of the transmission coefficient in the integration over the voltage bias window) 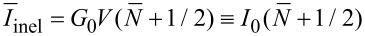 with


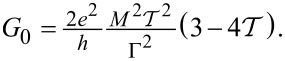


The second stage enables us to evaluate the noise [19] and higher order cumulants of the passed charge distribution by noticing that the above first-stage scheme can be slightly generalized to the cases in which also the amplitude of oscillations is a slow function of time *A*(*t*). In order to get a consistent theory the timescale of *A*(*t*) must be significantly slower than the oscillation period 2π/ω. This is satisfied for sufficiently weak coupling *M* such that the rates γ_↓_*_,_*_↑_ for the change of the occupation number 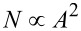 are much less than ω (this is true for small enough dimensionless coupling constant 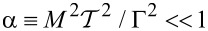) and the second stage of the coarse-graining, now for the oscillator *amplitude* (or, equivalently, occupation number) can be performed. Here, we assume that the instantaneous (on the long time scale 1/γ_↓_*_,_*_↑_) current is proportional (apart from the trivial constant term *G*_0_*V*/2 contributing additively just to the mean current, which will be ignored in the following) to the instantaneous occupation number *N*(*t*), i.e., *I*_inel_(*t*) = *I*_0_*N*(*t*). As a result we get for the cumulants of the electronic current for large voltages 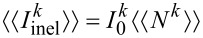 exclusively in terms of the (classical) dynamics of the oscillator occupation number.

The evolution of the occupation number probability density *P_n_*(*t*) that *N*(*t*) = *n* can be microscopically derived using the weak-coupling assumption from the secular Born–Markov approximation to the evolution equation for the reduced density matrix of the oscillator. This approach leads to a birth–death type of the master equation [19,27–29]

[8]
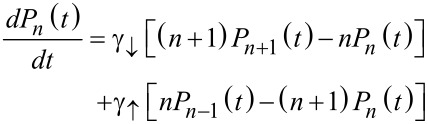


governed by the two microscopic parameters γ_↓_*_,_*_↑_ which are fully determined by the corresponding Fermi golden rule expressions relating them to the greater/lesser part of the non-equilibrium polarization operator of the vibrational mode [19]. Equivalently, they can be inferred from a more heuristic method of the energy power balance [10,12] applied to the dynamics of the mean occupation 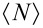 given by the simple linear rate equation 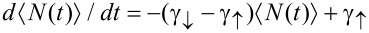. Comparison with the microscopic power balance equation [10,12] enables one to identify the inverse oscillator relaxation time τ^−1^≡γ_↓_ − γ_↑_ = 2αω/π and its stationary mean occupation number


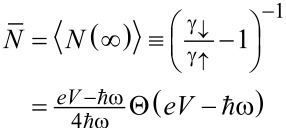


characterizing the geometric distribution


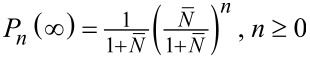


of the asymptotic state [19].

## Equation for the current cumulant generating function

The above master Equation 8 describes exclusively the state of the oscillator, but we are primarily interested in the electronic current statistics. The relation between the inelastic current cumulants and cumulants of the occupation number stated earlier could be used in principle for the evaluation of the current cumulants. However, the direct calculation of higher-order cumulants in the time domain is a rather complicated procedure. Instead, we can use a simple trick combining the master equation for the oscillator with the relation between the current and thus also passed charge 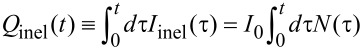. Since the charge *Q*_inel_(*t*) is a simple functional of the stochastic process *N*(*t*), we can write an extended master equation for the joint probability density *P_n_*(*q*,*t*) that *N*(*t*) = n and *Q*(*t*) = *q*. This reads (compare with, e.g., analogous approach in the context of work distributions [30])

[9]
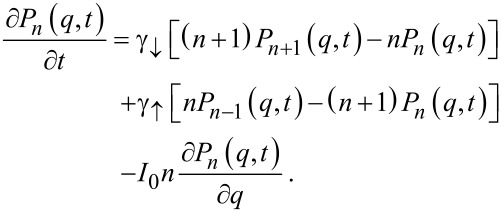


This equation can be recast into an equivalent form for the Laplace-transformed quantity


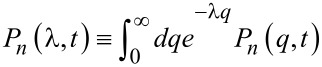


(here the charge *Q(t)* is due to the coarse-graining procedure considered as a continuous variable) more suitable for the direct evaluation of the current cumulant generating function (CGF)


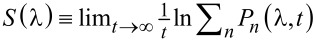


[10]
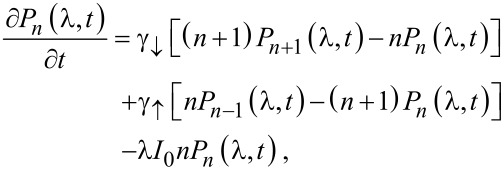


which can be solved by a *method of characteristics* [29]. For λ≠0 the generalized “probabilities” *P_n_*(λ,*t*) are not conserved, but that is the only difference from the procedure carried out in Section VI.6. of [29], which we outline here for the convenience of the reader. Introducing the moment generating function for the process *N(t)* via 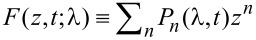 we can write

[11]
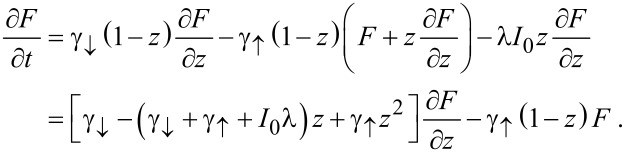


The equation for the characteristic curves reads

[12]
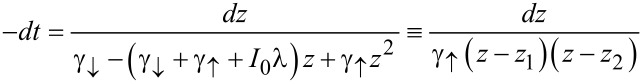


with the roots

[13]
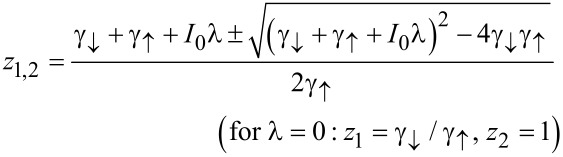


and has the solution

[14]
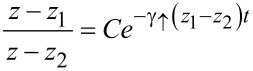


with an integration constant *C* labeling various characteristic curves. The variation of the function *F*(*z*,*t*;λ) along each separate characteristic curve is ruled by the equation

[15]
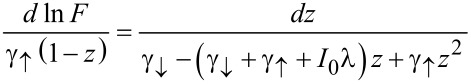


yielding

[16]
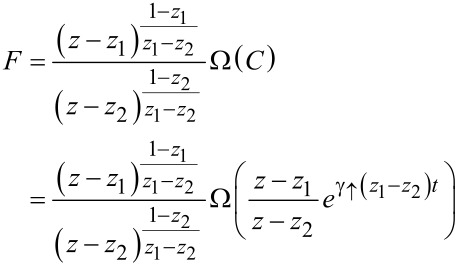


with Ω an arbitrary function of the integration constant *C*. It is fixed by the initial condition imposed on the probability distribution; since the stationary FCS of the current does not depend on initial conditions we are free to choose the most convenient one *P_n_*(*q*,*t* = 0) = δ_n,0_ δ(*q*) leading to the simplest expressions further on. The chosen initial condition implies the initial condition for *F*(*z*,*t* = 0;λ) = 1 and consequently

[17]
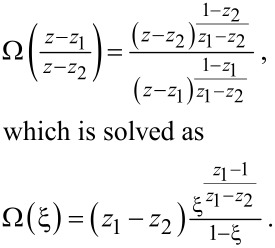


Putting things together we finally arrive at

[18]
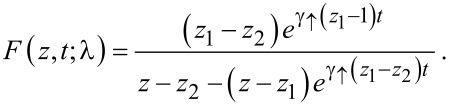


For λ = 0 we recover the solution (VI.6.4) of [29] with *r* = *m* = 0, *g* = 1, *a* = γ_↓_, *b* = γ_↑_. The large time asymptotics of Equation 18 is


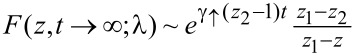


which leads to the sought-for expression for the CGF

[19]
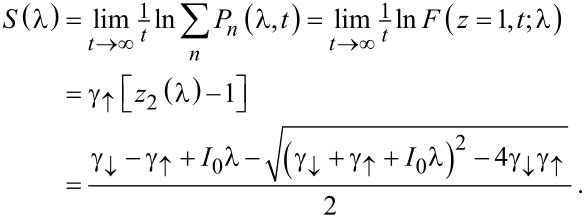


This is the main result of our paper, which allows us to analyze the behavior of the current statistics for large voltage, where the classical stochastic description is justified.

## Results and Discussion

Equation 19 yields for the mean current

[20]
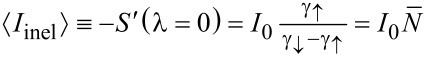


and the noise

[21]
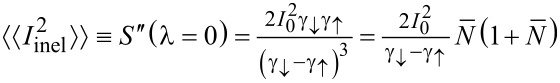


in accordance with physical intuition and previous results [19]. Apart from the offset *I*_0_/2 applicable just to the mean current, the cumulants are determined by the the square root expression


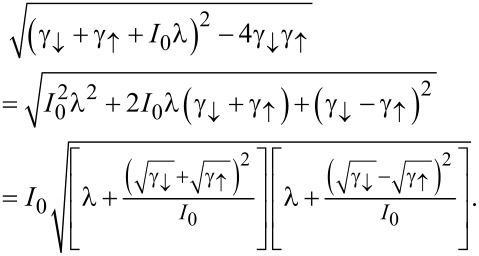


Here, we still restrict the discussion to the particular case of a single electronic level characterized by the elastic transmission 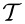 at zero temperature and the oscillator mode decoupled from any other degrees of freedom apart from the electronic level, i.e., with no external damping studied previously [18–20]. The above result (Equation 19) can be, however, applied under far wider conditions (multilevel dot, non-zero temperature and/or external damping of the oscillator mode) as we briefly discuss in the concluding section.

In the case specified above, we can obtain the large-*V* asymptotics of the CGF by identifying the known leading contributions of the constituent parts. From the definitions 
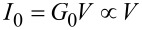
 and 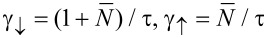 with the voltage-independent relaxation time and


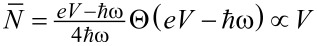


for large voltages the dominant contribution to the cumulants comes from the second square bracket under the square root as the term


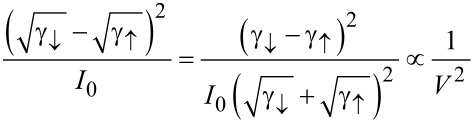


approaches zero with growing voltage while the offset in the other bracket saturates at a constant value. Therefore, the derivative at λ *= 0* is dominated [31] by the second bracket and we can safely put λ *= 0* in the first one as its derivatives are of a lower order in *V*. Collecting all factors we eventually obtain the formula for the asymptotic behavior of current cumulant generating function reading (with the dimensionless coupling constant 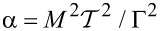)

[22]
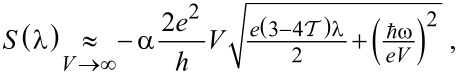


which leads to the asymptotic expressions for cumulants

[23]
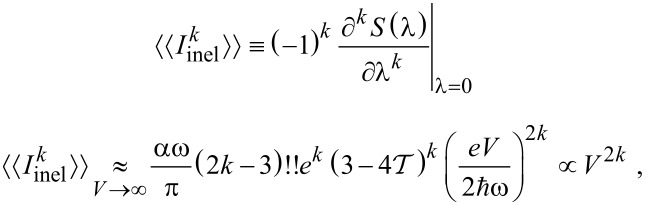


with 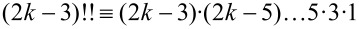. This factorial growth of high-order cumulants is a generic behavior [31–32]. With the extended definition (−1)!! ≡ 1, Equation 23 holds also for *k* = 1, i.e., for the mean current (the neglected offset *I*_0_/2 grows linearly in *V* and does not contribute to the leading asymptotics). These results agree with [26] for the quadratic behavior of the mean inelastic current and [19] for the quartic growth of the inelastic noise for the unequilibrated oscillator mode. Furthermore, Equation 22 is identical to the large-voltage asymptotics of the full microscopically derived CGF expression for the above specific model by Utsumi et al. [20] (for an explicit comparison notice that ours and Utsumi’s definitions of Γ differ by a factor of 2). However, these findings are at variance with Equation 15 of [18] predicting 
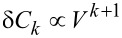
, which we thus must conclude to be incorrect.

## Conclusion

We have shown by a physically intuitive approach that the large-voltage asymptotics of electronic transport statistics through a nanosystem with weak electron-vibrational interaction can be determined from the classical stochastic dynamics of the vibrational occupation number. Equation 19 yielding the cumulant generating function is actually of general validity (not only for the specific single-level model explicitly studied throughout this text) with only three microscopic input parameters *I*_0_ and γ_↑_*_,_*_↓_ (or, alternatively, τ and 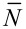) which must be evaluated for each model separately. Their general expressions are well known in the literature [10,12,19,25] and can/have been applied to cases with multiple electronic levels, finite temperatures, and/or external damping (whose magnitude can be even assessed from ab-initio calculations [33]). In particular, all the relevant quantities for the multilevel case corresponding to the general Hamiltonian introduced in the Model section treated in the wide band limit and with the account of the external oscillator damping are explicitly stated in the Supplement of [19] and will lead to a qualitatively similar behavior as the single level case discussed here.

Recently, the LOE method has been extended [34] beyond the wide-band approximation which assumes Γ *>> k*_B_*T*, 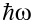, *eV* and has been used here as well. Finite bandwidth can have important consequences on the mode heating as was shown in [35] but also these effects can be actually captured within the present approach (at least in the regime Γ ≥ *eV >>* 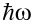 ensuring the proper time scales separation for the validity of the coarse-graining procedure) as long as the correct microscopic inputs for *I*_0_(*V*), γ_↑_(*V*), and γ_↓_(*V*) as functions of the voltage are provided into Equation 19. Even though there may not exist explicit analytical expressions for them in these more complicated cases, the present approach still offers huge level of simplification compared to the analogous full microscopic NEGF expressions for the cumulant generating function.

Finally, one may ask how relevant are these findings to the interpretation of experiments. Apart from the issue of validity of various underlying assumptions (such as the wide-band limit approximation), there is a question whether sufficiently high voltages can be realized in the experiment. Indeed, it is well known that for sufficiently large voltages (specific for a given experiment) the atomic/molecular junctions loose their structural stability and eventually break down. In this respect, the achievable “large-voltage” range may be fairly limited and, thus, our conclusions irrelevant. However, we believe that this is not the case. As we explicitly showed in [19] the large-voltage asymptotics for the inelastic noise is fully determined by the above approach in the dominant orders *V*^4^ and *V*^3^ and only in the order *V*^2^ there are deviations from the exact quantum-mechanical result, i.e., the relative error goes as 
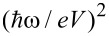
, which already for 
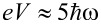
 is on the order of a few per cent. In the experiment (Figure 1 of [24]), 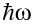 ≈ 20 meV, while the measurements are done easily up to a voltage of *V* = 80 mV without any traces of instability, i.e., they can be likely extended even higher. Thus, we are convinced that our large-voltage predictions are within the reach of the currently available experiments.
